# Trends and determinants of condomless sex in gonorrhoea patients diagnosed in France through the sentinel surveillance network ResIST, 2005–2014

**DOI:** 10.1186/s12889-020-09703-4

**Published:** 2020-10-28

**Authors:** Oche Adam Itodo, Delphine Viriot, Annie Velter, Lucie Leon, Nicolas Dupin, Beatrice Bercot, Agathe Goubard, François Lassau, Sébastien Fouere, Pervenche Martinet, William Tosini, Sophie Florence, Referents for the Regional Offices of the French National Public Health Agency (Santé Publique France), Lyderic Aubert, Emmanuel Belchior, Elise Brottet, Anne Etchevers, Aurélie Etienne, Bertrand Gagnière, Quiterie Mano, Esra Morvan, Bakhao N’Diaye, Nathalie Nicolay, Ronan Ollivier, Laurence Pascal, Annie-Claude Paty, Marie-Eve Raguenaud, Cyril Rousseau, Jeanine Stoll, Sabrina Tessier, Alexandra Thabuis, Jenifer Yai, Florence Lot, Ndeindo Ndeikoundam Ngangro

**Affiliations:** 1 Santé Publique France (The French National Public Health Agency), Saint Maurice, France; 2 French National Reference Centre for Bacterial STI (Syphilis), APHP, Cochin University Hospital, Paris, France; 3 French National Reference Centre for Bacterial STI (Gonorhoae), APHP, Saint Louis University Hospital, Paris, France; 4 Alfred Fournier Institute, Paris, France; 5 Dermatology Department, AP-HP, Saint-Louis Hospital, Paris, France; 6 Departmental Committee of Seine Saint Denis, Bobigny, France; 7 Departmental Committee of Bouches-du-Rhônes, Marseille, France; 8 STI Clinic (CeGIDD), Alfred Fournier Institute, Paris, France; 9 Sexual Health Centre Figuier, Paris, France

**Keywords:** Gonorrhoea, France, MSM, Surveillance, Sexual behaviour

## Abstract

**Background:**

Gonorrhoea is increasing in France since its resurgence in the late 1990’s. Understanding trends of condomless sex is a requirement to tailor prevention toward most exposed individuals. This study aims to analyse trends and determinants of condomless penetrative sex (PS) in MSM and heterosexuals diagnosed with gonorrhoea in France.

**Methods:**

A standardized self-administered questionnaire filled by 3453 patients was used to monitor condomless sex through the sentinel surveillance network ResIST between 2005 and 2014. Trends were used to describe consistent condom use for penetrative sex (PS). A logistic regression model analysed patients’ characteristics associated with condomless PS.

**Results:**

Between 2005 and 2014, condomless PS increased regardless of sexual orientation. Condomless PS was particularly common among HIV positive men who have sex with men (MSM (65%)). People living in metropolitan regions outside Paris area (adjusted odds-ratio (AOR) [95% CI] =1.33[1.12–1.58]) were more likely to engage in condomless PS. Conversely, MSM (AOR [95% CI] =0.21 [0.16–0.29]), HIV seronegative patients (AOR [95% CI] =0.68 [0.51–0.89]), patients diagnosed in hospital (AOR [95% CI] = 0.66 [0.45–0.97]) and multi-partners (≥ 10 partners, AOR [95% CI] = 0.54 [0.40–0.74]) were more likely to use condoms.

**Conclusions:**

These findings highlight a decreasing use of condom in MSM and heterosexuals diagnosed with gonorrhoea. Prevention strategies should take in account drivers of condomless sex in a context of uncontrolled STI epidemics.

## Background

In France, gonorrhoea has continued to rise since its resurgence in the late 1990’s [1]. Considering its complications such as salpingitis, ectopic pregnancy, pelvic inflammatory disease, infertility, its role in HIV transmission and the threat of antibiotic resistance, gonorrhoea is considered a public health concern [2–5]. Moreover, asymptomatic infections contribute to its spread.

In France, 15,100 cases were biologically confirmed in 2012 with a rate of 39 per 100,000 for people aged 15 to 59 years [6]. National surveillance data show that gonorrhoea primarily spread between men who have sex with men (MSM) [1].

With its short incubation of 2 to 5 days [2], gonorrhoea diagnosis trends could serve as a proxy to analyse changes in sexual behaviour and sexually transmitted infection (STI) prevention. Thus, its spread probably reflects increasing condomless sex described by behavioural studies among MSM [7–13]. Nevertheless, to our knowledge, no study purposely analysed determinants of condom use in patients diagnosed with STI in France, particularly in heterosexuals. This study aims to analyse trends and determinants of condomless penetrative sex (PS) in MSM and heterosexuals diagnosed with gonorrhoea, by employing for the first time the continuous data of the national surveillance of gonorrhoea in France [1].

## Methods

### Setting

In France, the national surveillance of gonorrhoea relies on a sentinel network of clinicians, “ResIST”, collecting demographic, clinical, biological and behavioural data [1]. During the study period (2005–2014), reported cases were mostly (99.6%) diagnosed by physician working in free STI clinics (named CeGIDD in France) [1]. These facilities target mainly high risk groups and disadvantaged ones such as MSM and migrants from high incidence countries, while general population might visit primarily general practitioners or non-hospital specialists for STI concerns. Almost all reported cases were diagnosed in mainland regions, as the ResIST network participation is largely insufficient in the overseas regions excluded from these analyses [1]. The network has 116 STI clinics, 14 hospital services and 594 clinicians in 2018. In 2012, ResIST was estimated to cover 6.3% of gonorrhoea diagnoses in France [6].

### Case definition

Gonorrhoea cases were bacteriologically defined through culture or nucleic acid amplification testing (NAAT) regardless anatomical location.

### Study population

Gonorrhoea patients reported to the surveillance network between 2005 and 2014 and who completed a self-administered structured behavioural questionnaire were the studied population.

### Data collection

Doctors filled a questionnaire with demographic, clinical and biological information. Patients completed a structured questionnaire with information related to their sexual practices within the previous 12 months.

### Study variables

Based on their reported sex and sexual practices, patients were categorized into groups of transmission; men who have sex with men (MSM), men who have sex with women exclusively (MSW), women who have sex with women (WSW) and women who have sex with men exclusively (WSM). Only one WSW was identified and subsequently removed from analyses. We classified the number of sexual partners in the last 12 months into sections (1 partner, 2 to 9 partners, 10 or more partners), as well as age ([14–20 years], [20–30 years], [30–40 years], [40–50 years], [50–60 years], and 60 years or over). Having steady (at least two sexual contact) or casual partner(s) (one sexual contact) in the last 12 months, knowledge about partners HIV status (positive, negative or unknown) for the last casual partner and all steady partners in last 12 months, HIV co-infection (positive, negative or unknown) were all variables of interest. Condomless sex defined as at least a condomless intercourse in the last 12 months, was ascertained for anal, vaginal and oral sex.

### Statistical analyses

Patients’ characteristics and sexual behaviour were described using proportions and median, for categorical and continuous variables respectively. Trends were used to analyse change across time, considering diagnosis centres reporting at least one case yearly over the last 3 years to take in account the fluctuation of centres’ participation in ResIST [1]. Then quantitative variables were compared using the non-parametric Wilcoxon and Kruskal-Wallis tests whereas categorical variables were compared using the Fisher’s exact test to assess association.

Using condomless penetrative sex whether anal or vaginal (PS) as an outcome, we carried out univariate and bivariate analyses after adjusting for the sexual orientation (MSM versus MSW and WSM) and HIV status since surveillance data demonstrated substantial differences in the way sexually transmitted infections (STI) spread considering these determinants [1]. Then, a logistic regression model was fitted to assess association between condomless PS and variables identified through the uni/bivariate analyses. All variables with *p* ≤ 0.20 were included in the model and backward selected according to the Hosmer and Lemeshow approach. The significance level was set at *p* < 0.05. Interactions (sexual orientation and HIV status) were tested in order to expand understanding of the relationships among variables but results were not included. Age was incorporated using fractional polynomials. Missing data were preserved during all analyses performed with Stata 12.1.

### Ethical approval

ResIST network was approved by the French Personal Data Protection Authority (CNIL authorization 902,057). “All procedures performed in studies involving human participants were in accordance with the ethical standards of the institutional and/or national research committee and with the 1964 Helsinki declaration and its later amendments or comparable ethical standards.”

### Informed consent

“Informed consent was obtained from all individual participants included in the study.”

## Results

### Population

**Fig. 1:**
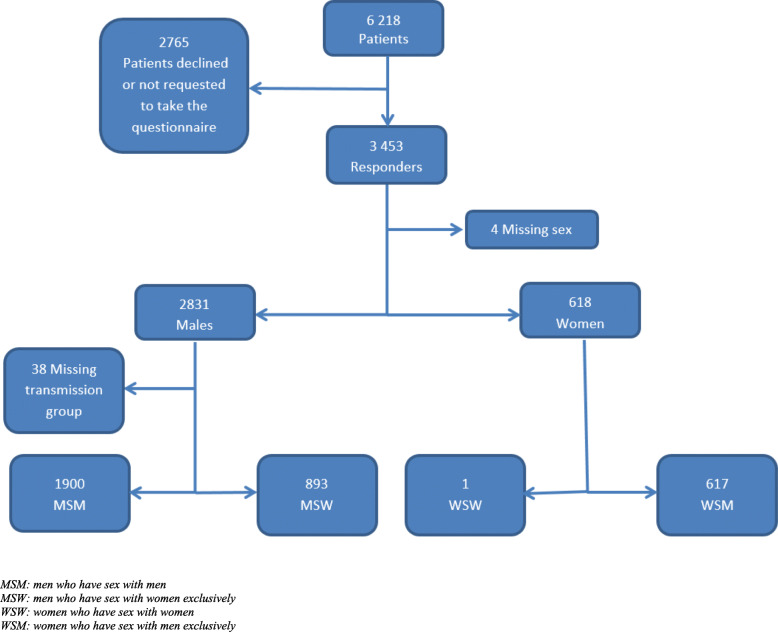
Gonorrhea patients’ characteristics, ResIST network 2005–2014, France

Between 2005 and 2014, 3453 (66.0%) patients reported with gonorrhoea completed a self-administered behavioural questionnaire (Fig. 1). They were more frequently men (82% versus 80%, *p* = 0.038), MSM (55.7% versus 43%, *p* < 0.001) and diagnosed in hospitals (3.9% versus 1%, *p* < 0.001), compared to patients who did not fill this questionnaire. During the study period, the proportion of women, notably WSM, increased from 3.3 to 21% among patients reported to the ResIST network. The proportion of 40–50 years olds decreased from 15 to 9%. The proportion of of people living with HIV remains steady over the time as 12%.

There were 26.2% MSW and 18.1% WSM among participants. Median age for MSM (28 years) patients was higher than for heterosexual males (25 years) and heterosexual females (21 years) (*p* < 0.0001). People aged between 20 and 30 years represented more than half of the study population (Table 1). The majority (76.2%) of the patients were born in France. Most patients were symptomatic at the time of gonorrhoea diagnosis: heterosexual men (90.0%), MSM (71.2%) and heterosexual females (53.3%) (*p* < 0.001). STI symptoms were the main motivation for seeking healthcare (67.5% in MSM, 85.2% in heterosexual men and 34.2% in heterosexual women) while suspected STI in a sexual partner and routine testing were frequently reported by women (21.4 and 24.2%) compared to men (respectively 8.9 and 12% MSM and 4.4 and 4.6% heterosexual men, *p* < 0.001). HIV-coinfection level remained high for MSM (13.9% versus 2.3% heterosexual men and 0.2% heterosexual women, *p* < 0.001).

**Table 1:** Characteristics of respondent gonorrhoea patients to the behavioral questionnaire by transmission groups. ResIST network 2005–2014, France

	MSM ***N*** = 1900	MSW ***N*** = 893	WSM ***N*** = 616
**Age class (%)**			
14–20 years	103 (5.4)	80 (9.0)	185 (30)
20–30 years	949 (49.9)	544 (60.9)	351 (57)
30–40 years	488 (25.7)	149 (16.7)	57 (9.3)
40–50 years	234 (12.3)	62 (6.9)	14 (2.3)
50–60 years	81 (4.3)	26 (2.9)	4 (0.6)
60+ years	31 (1.6)	17 (1.9)	0 (0.0)
Missing	14 (0.7)	15 (1.7)	5 (0.8)
**Region of residence** (%)			
Paris region	695 (36.6)	268 (30.0)	118 (19.2)
Other metropolitan regions	1109 (58.4)	540 (60.5)	454 (73.7)
Missing	96 (5.1)	85 (9.5)	44 (7.1)
**Country of birth** (%)			
France	1510 (79.5)	603 (67.5)	487 (79.1)
Other European countries	98 (5.2)	19 (2.1)	19 (3.1)
Non-European countries	209 (11.0)	223 (25.0)	84 (13.6)
Missing	83 (4.4)	48 (5.4)	26 (4.2)
**Motive for consultation (%)**			
Genital signs of STI	1283 (67.5)	760 (85.1)	210 (34.1)
Partner with STI	169 (8.9)	39 (4.4)	132 (21.4)
Routine testing	227 (11.9)	41 (4.6)	149 (24.2)
Other clinical signs	62 (3.3)	11 (1.2)	38 (6.2)
Missing	159 (8.4)	42 (4.7)	87 (14.1)
**Presence of symptoms (%)**			
Yes	1352 (71.2)	803 (90.0)	328 (53.2)
No	545 (28.7)	87 (9.7)	285 (46.3)
Missing	3 (0.2)	3 (0.3)	3 (0.5)
**HIV serologic status** (%)			
Newly diagnosed	20 (1.1)	7 (0.8)	1 (0.2)
Known seropositivity	244 (12.8)	13 (1.5)	0 (0.0)
Negative	1482 (78.0)	766 (85.8)	556 (90.3)
Missing	154 (8.1)	107 (12.0)	59 (9.6)

*MSM* men who have sex with men, *MSW* men who have sex with women exclusively, *WSM* women who have sex with men exclusively

### Sexual behaviours

In the last 12 months preceding the gonorrhoea diagnosis, MSM reported a median number of 10 sexual partners which remained steady during the surveillance period (Table 2). A median of 4 sexual partners was observed for heterosexual males and females within this same period. A higher proportion of heterosexual patients (70.7% of men and 81.9% of women versus 63.2% of MSM) reported steady partner(s) and majority of heterosexual men (79.7%) and MSM (89.4%) had casual partner(s) compared to heterosexual women (49.6%). Quarter of heterosexual women (28.4%) and 15.8% heterosexual men were unaware of their steady partners HIV status compared to a lower proportion of MSM (6.5%). Conversely, majority of MSM (56.7%) and heterosexual men (51.5%) were unaware of their casual partners HIV status. Half of heterosexual women did not answer this question while 29.2% also ignore casual partner HIV status.

**Table 2:** Sexual behaviours of patients diagnosed with gonorrhoea in the last 12 months. ResIST network 2005–2014, France

	MSM	MSW	WSM
**Number of partners (%)**			
1 partner	132 (7.0)	121 (13.6)	157 (25.5)
2 to 9 partners	748 (39.4)	537 (59.5)	403 (64.8)
10 partners or more	924 (49.2)	185 (21.4)	37 (6.7)
Missing	86 (4.53)	50 (5.6)	19 (3.1)
**Systematic condom use for anal sex (%)**			
Yes	701 (36.9)	166 (18.6)	26 (4.2)
No	1154 (60.7)	356 (39.9)	171 (27.8)
Missing	45 (2.4)	371 (41.5)	419 (68.0)
**Systematic condom use for vaginal sex (%)**			
Yes		159 (17.8)	48 (7.8)
No	N.A	690 (77.3)	544 (88.3)
Missing		44 (4.9)	24 (3.9)
**Systematic condom use for oral sex (%)**			
Yes	24 (1.3)	21 (2.4)	9 (1.5)
No	1798 (94.6)	737 (82.5)	446 (72.4)
Missing	78 (4.1)	135 (15.1)	161 (26.1)
**Steady partner(s) (%)**			
Yes	1200 (63.2)	631 (70.7)	505 (82.0)
No	682 (35.9)	262 (29.3)	105 (17.0)
Missing	18 (0.9)	0 (0.0)	6 (1.0)
**HIV status of steady partner(s) (%)**			
Positive	117 (6.2)	9 (1.0)	1 (0.2)
Negative	941 (49.5)	464 (52.0)	314 (51.0)
Not known	123 (6.5)	141 (15.8)	175 (28.4)
Missing	719 (37.8)	279 (31.2)	126 (20.5)
**Condom use with steady partner(s) for previous PS (%)**			
Yes	477 (25.1)	191 (21.4)	138 (22.4)
No	628 (33.1)	391 (43.8)	346 (56.2)
Missing	795 (41.8)	311 (34.8)	132 (21.4)
**Condom use with steady partner(s) for previous oral sex (%)**			
Yes	68 (3.6)	45 (5.0)	18 (2.9)
No	1095 (57.6)	484 (54.2)	344 (55.8)
Missing	737 (38.8)	364 (40.8)	254 (41.2)
**Casual partner(s) (%)**			
Yes	1698 (89.4)	712 (79.7)	305 (49.5)
No	189 (9.9)	178 (19.9)	305 (49.5)
Missing	13 (0.7)	3 (0.3)	6 (1.0)
**Knowledge of HIV status of the last casual partner (%)**			
Positive	78 (4.1)	9 (1.0)	1 (0.2)
Negative	517 (27.2)	226 (25.3)	117 (19.0)
Not known	1077 (56.7)	460 (51.5)	180 (29.2)
Missing	228 (12.0)	198 (22.2)	318 (51.6)
**Condom use with casual partner(s) for the last PS (%)**			
Yes	1160 (61.1)	358 (40.1)	172 (27.9)
No	334 (17.6)	299 (33.5)	121 (19.6)
Missing	406 (21.4)	236 (26.4)	323 (52.4)
**Condom use with the casual partner(s) for the last oral sex (%)**			
Yes	111 (5.8)	71 (8.0)	32 (5.2)
No	1527 (80.4)	524 (58.7)	179 (29.1)
Missing	262 (13.8)	298 (33.4)	405 (65.7)

*MSM* men who have sex with men, *MSW* men who have sex with women exclusively, *WSM* women who have sex with men exclusively

**Fig. 2:**
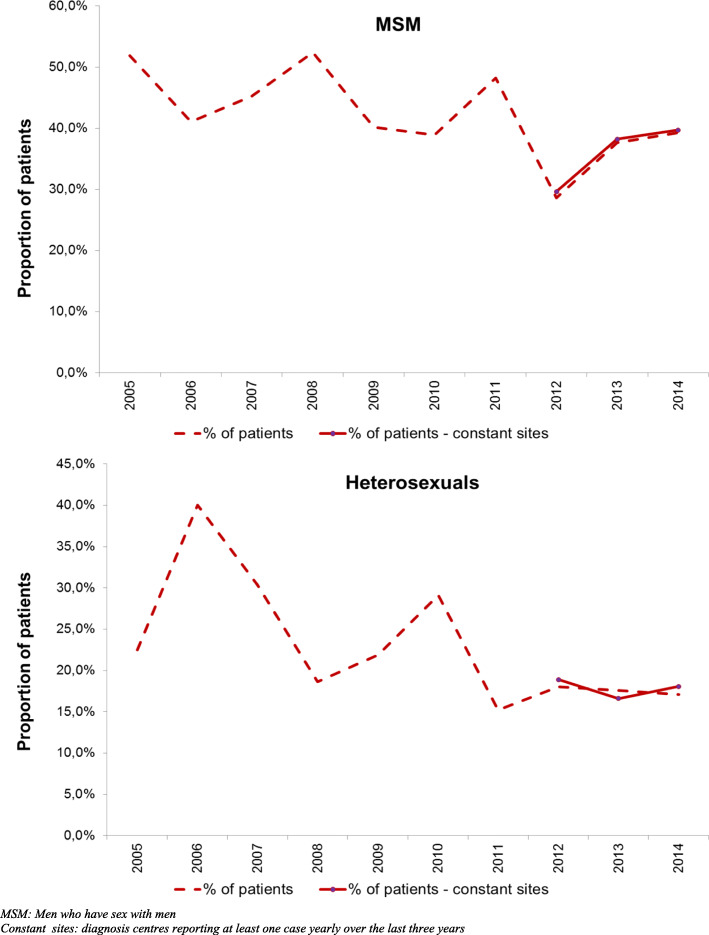
Proportion of gonorrhea patients consistently used condom for penetrative sex by sexual orientation. ResIST network 2005–2014, France

Between 2005 and 2014, a significant decline in the consistant use of condoms for PS was observed whether patients were MSM (from 51.9 to 39.3%, *p* < 0.001) or heterosexuals (from 22.5 to 17.1%, *p* = 0.005) despite a slight increase observed from 2012 to 2014 in MSM and from 2013 to 2014 in heterosexuals (Fig. 2). Condom use for PS with casual (61.1%) or steady (25.1%) partner(s) remained higher among MSM compared to heterosexuals (respectively 40.1 and 21.4% men, 28.0 and 22.4% women, *p* < 0.01) during the study period (Table 2). Nevertheless, considering specifically anal sex, 60.7% of MSM did not use condoms systematically (*p* < 0.01). Majority of heterosexuals (77.3% men and 88.3% women) did not consistently protect their vaginal intercourse (*p* = 0.001).

Furthermore, condom use for oral sex with casual and steady partners was not frequent, whether patients were MSM (5.8 and 3.6%), MSW (8.0 and 5.0%) or WSM (5.2 and 2.9%). A higher proportion of MSM reported no protection for their most recent oral sex with casual (80.4%) partner(s) (Table 2).

### Patient’s characteristics associated with condom use

The multivariable model (Table 3) shows that condomless sex was significantly less frequent among patients aged 30–50 years (adjusted odds-ratio (AOR) [95% confidence interval (95% CI)] =0.51 [0.36–0.71] for 40–50 year olds). People living in metropolitan regions outside Paris area (AOR [95% CI] =1.33 [1.12–1.58]) were more likely to engage in condomless PS. Condomless PS was common in patients diagnosed with gonorrhoea after 2011 (AOR [95% CI] = 1.61 [1.25–2.09] in 2012) compared to patients diagnosed before. Conversely, condomless PS was less common among MSM (AOR [95% CI] =0.21[0.16–0.29]), MSW (AOR [95%CI] = 0.40[0.29–0.55]), HIV negative patients (AOR [95% CI] =0.68 [0.51–0.89]) and patients with unknown HIV status ([AOR [95% CI] =0.63 [0.44–0.90]). Condomless PS was also less usual in patients reporting multiple sexual partners (at least 10 partners in last 12 months: AOR [95% CI] = 0.54 [0.40–0.74]).

**Table 3:** Sexual and sociodemographic factors associated with condomless penetrative sex in gonorrhea patients. ResIST network 2005–2014, France

	Univariate analysis			Univariate analysis for MSM			Univariate analysis for MSW			Univariate analysisfor WSM			Logistic Regression
	Protected PS n (%)	Condomless PS n (%)	p	Protected PS n (%)	Condomless PS n (%)	P	Protected PS n (%)	Condomless PS n (%)	P	Protected PS n (%)	Condomless PS n (%)	p	AOR (95%)
**Sex**													
Men	998 (35.9)	1775 (64.1)	< 0.001										
Women	65 (10.6)	548 (89.4)											
Missing	0 (0)	1 (100)											
**Sexual orientation in the last 12 months**													
WSM	63 (10.3)	546 (89.7)	< 0.0001										1
MSW	232 (26.3)	649 (73.7)											0.40 (0.29–0.55)
MSM	760 (40.8)	1104 (59.2)											**0.21 (0.16–0.29)**
Missing	7 (21.9)	25 (78.1)											**0.54 (0.22–1.31)**
**Age**													
14–19 years	71 (19.4)	295 (80.6)	< 0.0001	39 (38,2)	63 (61.8)	0.077	18 (22.5)	62 (77.5)	0.108	13 (7.2)	168 (92.8)	0.05	1
20–29 years	526 (28.6)	1314 (71.4)		355 (37.9)	581 (62.1)		132 (24.5)	407 (75.5)		36 (10.3)	313 (89.7)		0.80 (0.62–1.02)
30–39 years	266 (38.6)	423 (61.4)		209 (43.6)	270 (56.4)		44 (30.1)	102 (69.9)		11 (19.3)	46 (80.7)		**0.64 (0.48–0.85)**
40–49 years	137 (44.8)	169 (55.2)		119 (52.2)	109 (47.8)		25 (41.0)	36 (59.0)		3 (21.4)	11 (78.6)		**0.51 (0.36–0.71)**
50–59 years	39 (35.8)	70 (64.2)		30 (38.4)	48 (61.5)		8 (30.8)	18 (69.2)		3 (75.0)	1 (25.0)		0.81 (0.49–1.33)
60 + years	18 (40.0)	27 (60.0)		14 (50.0)	14 (50.0)		3 (18.1)	13 (81.3)		0 (0.0)	0 (0.0)		0.62 (0.30–1.25)
Missing	6 (18.7)	26 (81.3)		4 (30.8)	9 (69.2)		2 (15.4)	11 (84.6)		0 (0 .0)	5 (100.0)		1.43 (0.56–3.67)
**Region of residence**													
Paris region	408 (38.1)	662 (61.9)	< 0.0001	311 (45.9)	367 (54.1)	< 0.001	79 (29.8)	186 (70.2)	0.30	15 (12.8)	102 (87.2)	0.31	1
Other metropolitan regions	589 (28.2)	1503 (71.8)		407 (37.2)	687 (62.8)		131 (24.7)	400 (75.3)		46 (10.3)	402 (89.7)		**1.33 (1.12–1.58)**
Missing	66 (29.3)	159 (70.7)		42 (45.7)	50 (54.3)		22 (25.9)	63 (74.1)		2 (95.4)	42 (4.6)		**1.15 (0.83–1.60)**
**Consultation sites**													
Free STI clinics	1007 (31.1)	2232 (68.9)	0.165	716 (40.6)	1047 (59.4)	0.62	221 (25.9)	632 (74.1)	0.20	62 (10.5)	528 (89.5)	0.18	1
Hospital	53 (38.7)	84 (61.3)		43 (44.3)	54 (55;6)		10 (41.7)	14 (58.3)		0 (0)	16 (0)		**0.66 (0.45–0.97)**
Missing	3 (27.3)	8 (72.7)		1 (25.0)	3 (75.0)		1 (25.0)	3 (75.0)		1 (33.3]	2 (66.7]		**0.82 (0.21–3.30)**
**Year**													
2005–2011	467 (35.9)	835 (64.1)	< 0.0001	330 (46.6)	378 (53.4)	< 0.0001	114 (27.1)	306 (72.9)	0.71	19 (12.1)	138 (87.9)	0.14	1
2012	105 (23.6)	340 (76.4)		65 (29.3)	157 (70.7)		36 (29.0)	88 (71.0)		4 (4.2)	91 (95.8)		**1.61 (1.25–2.09)**
2013	190 (28.9)	467 (71.1)		132 (38.1)	214 (61.9)		36 (23.7)	116 (76.3)		18 (12.0)	132 (88.0)		1.17 (0.94–1.46)
2014	301 (30.6)	682 (69.4)		233 (39.6)	355 (60.4)		46 (24.9)	139 (75.1)		22 (10.6)	185 (89.4)		**1.22 (1.01–1.48)**
**HIV status**													
Positive	100 (35.8)	179 (64.2)	0.199	90 (35.2)	166 (64.8)	0.14	10 (50)	10 (50)	0.032	0 (0.0)	1 (100)	0.44	1
Negative	860 (30.8)	1932 (69.2)		607 (41.6)	852 (58.4)		190 (25.2)	565 (74.8)		55 (10.0)	495 (90.0)		**0.68 (0.51–0.89)**
Missing	103 (32.6)	213 (67.4)		63 (42.3)	86 (57;7)		32 (30.2)	74 (69.8)		8 (13.6)	51 (86.4)		**0.63 (0.44–0.90)**
**Number of partners in the last 12 months**													
1 partner	66 (16.5)	333 (83.5)	< 0.0001	36 (28.4)	91 (71.6)	0.004	19 (16.4)	97 (83.6)	0.03	11 (7.2)	142 (92.8)	0.003	1
2 to 9 partners	513 (30.2)	1184 (69.8)		329 (44.2)	415 (55.8)		142 (26.6)	391 (73.4)		38 (9.5)	362 (90.5)		**0.52 (0.39–0.70)**
10 partners or more	435 (38.3)	702 (61.7)		366 (40.2)	544 (59.8)		56 (30.3)	129 (69.7)		10 (27.0)	27 (73.0)		**0.54 (0.40–0.74)**
Missing	49 (31.8)	105 (68.2)		29 (34.9)	54 (65.1)		15 (31.9)	32 (68.1)		4 (21.1)	15 (78.9)		**0.55 (0.35–0.86)**
**Steady partner(s) in the last 12 months**													
No	386 (36.5)	671 (63.5)	< 0.0001	294 (43.9)	375 (56.1)	0.008	73 (28.1)	187 (71.9)	0.45	13 (12.5)	91 (87.5)	0.62	
Yes	671 (28.9)	1647 (71.1)		461 (38.8)	728 (6.8)		159 (25.6)	462 (74.4)		50 (10.0)	452 (90.0)		
Missing	6 (50.0)	6 (50.0)		5 (83.3)	1 (16.7)		_	_		0 (0)	3 (100.0)		
**Casual partner(s) in the last 12 months**													
No	117 (17.5)	553 (82.5)	< 0.0001	55 (29.4)	132 (70.6)	0.0009	38 (22.2)	133 (77.8)	0.34	23 (7.6)	279 (92.4)	0.06	
Yes	942 (34.8)	1762 (65.2)		703 (42.0)	971 (57.0)		193 (27.3)	514 (72.7)		40 (13.1)	265 (89.9)		
Missing	4 (30.8)	9 (69.2)		2 (66.7)	1 (33.3)		1 (33.3)	2 (66.7)		0 (0)	2 (100)		
**Knowledge of the HIV status of steady partner(s) in the last 12 months**													
No	554 (30.3)	1273 (69.7)	< 0.0001	400 (38.2)	648 (61.8)	0.032	118 (25.4)	346 (74.6)	0.75	35 (11.2)	278 (88.8)	0.65	
Yes	104 (23.6))	336 (76.4)		52 (42.6)	70 (57.4)		37 (26.2)	104 (73.8)		15 (9.6)	160 (91.4)		
Missing	405 (36.2)	715 (63.8)		308 (44.4)	386 (55.6]		77 (27.9)	199 (72.1)		13 (10.7)	108 (89.3)		
**Knowledge of HIV status of the last casual partner**													
No	623 (36.5)	1082 (63.5)	< 0.0001	215 (41.8)	300 (58.2)	< 0.0001	55 (24.4)	170 (75.6)	0.19	11 (9.4)	106 (90.6)	0.0036	
Yes	308 (32.5)	639 (67.5)		20 (27.0)	54 (73.0)		4 (44.4)	5 (55.6)		1 (100.0)	0 (0.0)		
Missing	132 (18.0)	603 (82.0)		750 (58.8)	525 (41.2)		173 (26.7)	474 (73.3)		440 (89.6)	51 (10.4)		

*AOR* adjusted odds-ratio, *95%CI* 95% confidence interval, *MSM* men who have sex with men, *MSW* men who have sex with women exclusively, *WSW* women who have sex with women exclusively, *PS* penetrative sex

## Discussion

### Condomless penetrative sex

The surveillance data reveal a significant decrease in the use of condoms for PS among MSM and heterosexuals diagnosed with gonorrhoea in France from 2005 to 2014. These trends are consistent with repeated behavioural studies demonstrating increases in condomless anal intercourse among MSM in France [1]. Increasing trends of condomless anal sex were also described in high-Income Countries (United States, Australia, United Kingdom) for MSM, but also heterosexuals [9–19]. Despite prevention effort, the overall proportion of individuals using consistently condoms for PS during the last 12 months remains unsatisfactory whether patients were MSM or heterosexuals during the study period.

### Condomless oral sex

Although oral sex is currently quite common in France, it remains mostly mostly unprotected for MSM, MSM and WSM [1, 12]. Furthermore, a decrease was observed in the use of condoms for oral sex. This insufficient use of condoms for oral sex has also been observed in other European countries, notably in the United Kingdom [20, 21]. Moreover, increasing frequency of condomless oral sex in France, especially among the younger generation [13], might become a concern considering the potential development of antimicrobial resistance (AMR) in the pharyngeal infections [5, 22].

### Condom use and HIV status

HIV positive and negative patients seemed to be more and more reluctant to use condoms for PS, whether they were MSM or heterosexual. The uptake of antiretroviral therapy (ARV) might have changed sexual behaviours, by lowering the fear of HIV acquisition or spreading the belief that HIV infection is curable. Repeated behavioural surveys conducted in France might reflect this shifting perceptions and attitudes towards HIV with increases in condomless anal intercourses among MSM, whether they were seropositive for HIV or not [9–12]. This is also observed in individuals seeking HIV post-exposure prophylaxis (PEP) between 2006 and 2011 in Paris [13]. Studies conducted in high incomes countries confirm the same trend for unprotect anal intercourse, notably in HIV-discordant partners [13, 16, 18, 19, 23, 24].

A potential effect of HIV pre-exposure prophylaxis (PrEP) was not analysed since PrEP was officially launched in France in 2016. Future years’ data might give an insight of its impact on the use of condom and epidemiology of gonorrhoea. Indeed, expanding the range of HIV prevention tools could paradoxally be associated with increasing STI diagnoses in its beneficiaries; but PrEP consultations remain opportunities for regular screenings in core populations and consequently prevention to STI transmission for people not using condom [18, 25, 26].

### Patients characteristics associated with condomless penetrative sex

The multivariate model demonstrates that gonorrhoea patients aged 14–20 years were more reluctant to use condom for PS compared to older ones, and especially the 30–50 years olds. Youth was already reported as a risk factor for condomless anal sex in MSM in France and in other European countries even if the level of condom use in general population remains higher among young people [11–13, 15, 19]. Nevertheless, these studies also reported declining trends in condom use for young population. Belonging to “post-AIDS (acquired immune-deficiency syndrome) generation and having an adventure-oriented sexuality” might explain the vulnerability of this population, notably during sexuality experimentation because of a less frightening perception of HIV infection thanks to antiretroviral and a better controlled epidemic compared to 1980s and 1990s situation [11–13, 15, 19]. Nevertheless, even if condom use was more frequent above 20 years old, the model also shows a relatively close level of condom use among patients aged 50–60 years and 20–40 years old. But the drivers of condom use might differ among generations and should be taken into account when designing preventive action [9, 10].

Condomless PS was not significantly associated with patiens’sex though it might reflect a gender based difficulty to negotiate condom use as well a mixed effect of anal and vaginal penetration in the definition of PS used in the multivariable analyses [14]. Indeed, a study conducted in France, showed that women were more likely to be engaged in condomless sex [12].

Condomless PS was also significantly reported by patients diagnosed in other mainland regions compared to Paris area. A lower concentration or access to sexual health services in remotes or rural areas might explain this situation as well as delays and inequalities in prevention expansion within the geographic areas. Indeed, gonorrhoea resurgence started in Paris area in the early 2000 and then spread towards others regions. Prevention might have followed the same geographic pattern.

Moreover, patients diagnosed in a hospital based facilities were more likely to use condom than those diagnosed in free STI clinics. These results confirm that highly exposed populations are probably well targeted by free STI clinics, where systematic and opportunistic testing are applied whether patients are symptomatic or not [1]. Under representation of patients diagnosed by general practitioners in the ResIST sentinel (0.4%) network probably hampered an accurate analysis of their behaviour. Cross sectional survey might give an insight of the situation in private medicine in France.

Gonorrhea diagnoses made after 2011 were significantly associated with condomless PS. It might reflect the rising trends of condomless anal penetration in MSM reported by behavioral studies in France [10, 11]. This prevention relapse is particularly concerning for HIV positive MSM, even if increasing risk was also reported by HIV negative MSM [10]. Nevertheless, the multivariate model demonstrates that patients seronegative for HIV were likely to use condom for PS. European results also demonstrate the same likelihood for seronegative MSM compared to seropositive ones, though HIV positive patients are mostly well sensitized [19]. Thus, retroviral treatments might shadow bacterial STI prevention among seropositive patients.

Multi-partnership over the last 12 months was associated with decreasing risk of condomless PS, suggesting a rising probability of using condom parallel to the number of sexual partners. Conversely, multi-partnership was reported in other contexts as a determinant of non-condom use. Qualitative studies conducted among STI patients or adjustment for additional sociodemographic variables (country of birth, education level, socioeconomic status …), behavioural information (sero-adataptive behaviours, insertive/receptive position …) and STI prevention/diagnosis access data (predisposing, enabling, needs and barriers factors) could enable further analyses.

### Limitations

STI surveillance relying on a sentinel surveillance network, these results are neither exhaustive nor representative of the French population but they reflects the behavioural trends of MSM and heterosexuals exposed to gonorrhoea, then infected and mostly detected in the mainland’s free STI clinics. Explored behaviours and determinants of condom use were limited by the surveillance constraints, notably the need to sustain a good level of participation. As gonorrhoea patients were almost exclusively reported by STI clinics (99%), a cross-sectional behavioural survey including private diagnostic centres, general practitioners and hospital units would give a complete picture of sexual behaviours trends in the general population, especially if health seeking behaviours, diagnosis delay, determinants of prevention use and additional behavioral information (i.e substance use ….) are collected. Moreover, reported patients are mainly symptomatic with frequent genital conditions, thus patients and especially MSM with asymptomatic pharyngeal and/or anal infections might need further behavioural analyses. Information on condom use for people that tested negative for gonorrhoea might help to monitor more accurately trends in condom use over time. Declarative information might over or underestimate some behaviours (e.g number of partner), because of social norms, desirability bias and recall bias. It was not possible to adjust analyses for repeated measurements per individual since anonymous identifier were not unique for patients and it was not possible to link patients throughout STI centers. Nevertheless, these surveillance data were the first to provide a continuous insight of sexual behaviours in highly exposed and infected MSM and heterosexual over the time.

## Conclusion

In a context of uncontrolled STI epidemic, these continuous behavioral data confirmed in exposed, then infected and diagnosed gonorrhoea patients, a decreasing likelihood of condom use [23, 24] regardless sexual orientation. Considering sexuality, changes in social norms, and prevention availability and accessibility, repeated behavioural surveys including any type of STI diagnostic centre might contribute to a better understanding of patients’ obstacles toward condom use and more largely difficulties to use prevention, compared to a standardized and continuous behavioural monitoring. Frequent screening remains the cornerstone to prevent STI transmission in core population not using condoms consistently.

## Data Availability

Access to the datasets used and/or analysed during the current study is restricted by the French Personal Data Protection Authority (CNIL authorization 902057). These data are available from the corresponding author on reasonable request.

## Abbreviations

AIDS: Acquired ImmunoDeficiency Syndrome
AMR: Antimicrobial resistance
AOR: Adjusted odds-ratio
ARV: Antiretroviral therapy
HIV: Human immunodeficiency virus
MSM: Men who have sex with men
MSW: Men who have sex with women exclusively
NAAT: Nucleic acid amplification testing
PEP: HIV post-exposure prophylaxis
PrEP: HIV pre-exposure prophylaxis
PS: Penetrative sex
STI: Sexually transmitted infections
WSW: Women who have sex with women exclusively
